# Leucocytosis in Appendicitis

**Published:** 1902-05-10

**Authors:** 


### LEUCOCYTOSIS IN APPENDICITIS.

In reference to this very important question as to the value of the indications given by the occurrence of leucocytosis, we cannot do better than refer to a recent article on leucocytosis as a point of prognosis in appendicitis by Dr. Henry M. Joy[1] and Dr. Frederick T. Wright From a careful observation of a considerable number of cases they conclude that the degree of leucocytosis is an index of the severity of the case; a high stationary or an increasing leucocytosis indicates a progressively serious case which demands immediate intervention ; a decreasing leucocytosis, save in some very few exceptional cases to be referred to again, points to a case of decreasing severity in which operation *may* be safely delayed, if for any reason it be so desired. It is interesting to observe that the authors fully recognise the danger of the quiescent period to which Mr. Tubby draws attention, for they say "Suppose, that in any given attack the usual signs and symptoms appear to indicate a marked diminution in its severity. If the first count be moderately high, say from 12,000 to 16,000, and the second or any subsequent count show a rise of, say, 2,000 or more, especially if there be an apparently continuous rise, we urge immediate operation *regardless of the fact that the clinical symptoms appear to show improvement.* In such an instance we operate solely on the basis of the leucocyte count regardless of all other indications, and our experience, as we shall show in detail, seems to justify us in so doing." The exceptional cases in which, with a lowered leucocytosis, the patient may still be going to the bad, they explain by saying that "it is only (speaking somewhat loosely) when an effort is being made by Nature to overcome disease that we find an increasing leucocytosis, and *per contra,* when from an ever-increasing toxæmia Nature is forced to give up the struggle, there may be a progressive fall in the blood count." But, as they properly say, in such a case of appendicitis, seen at a late stage with a progressively-falling leucocytosis, the general conditions would be such that even the inexperienced could not be misled. Excluding these cases, however, the authors are quite clear as to the utility and reliability of the indications given by the blood count, which on the one hand may indicate a morbid condition of increasing severity which demands operation, no matter what the clinical symptoms may be, and on the other may show that operation may be postponed with safety should such a course, for other reasons, appear desirable.

[1] New York Med. News, April 5.

